# Evaluation of family physicians’ anxiety during the COVID-19 process: provincial example from Turkey

**DOI:** 10.1186/s40359-022-01024-y

**Published:** 2022-12-17

**Authors:** Bahadir Yazicioglu, Muhammet Ali Oruc, Sule Ozdemir

**Affiliations:** 1 Department of Family Medicine, Samsun Education and Research Hospital, Samsun, Turkey; 2 Department of Family Medicine, Samsun University Faculty of Medicine, Samsun, Turkey; 3 Department of Public Health, Samsun University Faculty of Medicine, Samsun, Turkey

**Keywords:** Physicians, Primary care, Primary health care, Pandemics, Anxiety

## Abstract

**Background:**

In the natural progression of COVID-19, recovery usually takes months in most cases. Comprehensive evaluation of underlying complications requires a holistic approach as in primary health care, which creates additional workload and stress for family physicians.

**Methods:**

The descriptive-cross-sectional study was carried out in 226 family physicians in Samsun, Turkey. Ethical permissions were obtained to conduct the study. State-Trait Anxiety Inventory scale was used as data collection tool.

**Results:**

214 physicians were included in the study. The majority of the participants in the study were female. Most of the family physicians were not specialist physicians. Most of them were married. The majority of family physicians do not smoke and did not have any chronic diseases. The trait anxiety score of the physicians was 43.40 ± 8.50, and the situational anxiety score was 48.09 ± 11.55. The correlation between trait anxiety and situational anxiety was positive and significant. Gender difference did not make a significant difference on anxiety. Anxiety was significantly higher in patients with chronic disease. Marital status, having a child, and a history of COVID disease do not have a significant effect on anxiety.

**Conclusion:**

At the beginning of the COVID pandemic, the greatest struggle was given in secondary and tertiary healthcare institutions. In the following process, this burden shifted to primary health care institutions. This situation has increased the workload and stress of family physicians working in primary care. Therefore, it also increased perceived anxiety. Individuals with high trait anxiety scores have higher state anxiety scores.

## Introduction

Coronavirus Disease 2019 (COVID-19) is a disease caused by the Severe Acute Respiratory Syndrome-Coronavirus-2 (SARS-CoV-2) virus. It was first reported in Wuhan, China, in December 2019. The virus is highly contagious with its rapid contagion feature [1, 2]. As a result of the rapid spread in a short time, the World Health Organization (WHO) declared that COVID-19 had turned into a pandemic in March 2020 [3].

The emergence of a rapidly spreading and life-threatening new disease, causes significant pressure and burden on health workers and health systems [4]. To cope with the additional burden of the pandemic worldwide, a complete restructuring of all health systems services is required. In the early part of this pandemic, the focus in healthcare has shifted to differential diagnosis triage and COVID-19 management. Specialized COVID-19 centers have been established for follow-up and treatment. Due to the restructuring of the healthcare system to combat COVID-19, non-COVID patient admissions to secondary and tertiary hospitals have decreased. Primary health care services are more exposed to the follow-up of chronic diseases and therefore they are under a heavier workload [5]. At the same time, family physicians were at the forefront in the fight against the pandemic, as the first point of contact for the undifferentiated patient. In the natural course of COVID-19 in diagnosed patients, in most cases the first period appears to be a gradual improvement [5, 6]. However, full recovery is usually within months [7]. The long recovery period and the occurrence of serious complications in some patients reveal the importance of a comprehensive and holistic approach in primary health care [5, 6].

Many studies have shown that pandemics affect the psychological state of society. Repeated curfews, isolation periods at home after contact with infected people, social distance practices and fear of being infected with COVID-19 cause stress and anxiety in people. Health workers who are at the forefront of the fight against COVID-19 also have negative effects on mental health. Because healthcare workers are in close contact with infected patients, the risk of getting sick during pandemics is higher than the general population, and they have a heavy workload that can affect their psychological state [8, 9]. The increased workload, physical fatigue, lack of personal protective equipment, possible hospital-acquired contamination, and the necessity to make ethically difficult decisions such as prioritizing health care have dramatic effects on the physical and mental health of healthcare professionals [4].

Anxiety is an organic response characterized by increased concern due to uncertain dangerous situations or potential threats to the integrity of the organism [10]. Anxiety is the most common psychiatric disorder [11]. The global prevalence is approximately 16% [12]. Anxiety disorders are predicted to have a higher prevalence than detected and therefore are thought to cause more adverse effects and disability than chronic diseases [13]. The relationship between personal characteristics and psychological states may differ, and this has long been known in the medical sciences [14]. The most well-known of these distinctions are state anxiety and trait anxiety. These two types of anxiety are two related but distinct components [15]. State anxiety is a person’s perception of their current situation as dangerous and threatening. Its level increases in stressful situations and decreases as the stress disappears. Trait anxiety describes a person’s predisposition to experience anxiety. It is considered a personal trait. In trait anxiety, the anxiety is more intense and continuous [16].

In addition to the acceptance that therapeutic services are mostly concentrated in inpatient treatment institutions and intensive care units in the COVID pandemic, it is desired to draw attention to the effective provision of primary health care services. The primary health care service, which was at the forefront of the pandemic by welcoming undifferentiated patients, also faced protracted complications in the course of the pandemic. This situation has caused increased workload and stress in primary care physicians. From this point of view, “What is the anxiety caused by the pandemic on family physicians in primary health care services?” an answer to the research question was sought. During the pandemic process, it has been observed that studies on secondary and tertiary health care services related to the pandemic have been carried out. In this study, it was aimed to investigate the perceived anxiety of family physicians during the pandemic, the factors affecting the perceived anxiety level, and the relationship between personality structure and perceived anxiety level.

## Method

### Place of study

This study was carried out in Samsun, a coastal city in northern Turkey’s Central Black Sea region. The population of Samsun province in 2020 was 1,356,079 [17]. In Samsun, primary health care services are provided by 412 Family Physicians working in 141 Family Health Centers throughout the province. Population-based primary health care services are provided in Family Health Centers in all provinces of Turkey. About 3300 people are registered with a Family Physician. The number of registered persons per family physician shows a similar ratio across Turkey and creates a similar workload for primary health care services [18].

### Sampling and sample size

The population of this descriptive-cross-sectional and analytical study consists of 412 family physicians working in Samsun. Sample calculation was done with the G*Power program. The magnitude of the effect calculated according to previous studies [19] was calculated considering 0.24, the working power of 95%, type 1 error of 0.05. The minimum sample size was found to be 189 people. Considering the lack of data and questionnaires that may contain inappropriate answers (such as multiple choice markings or incorrect demographic data entry), a questionnaire was administered to 226 family physicians who volunteered to participate in the study.

226 family physicians working throughout the province of Samsun were selected by simple random method and a questionnaire was applied to the selected family physicians. While the data were processed into the statistical program, 12 questionnaires containing missing data and inappropriate were excluded from the study. The study was carried out with 214 questionnaires.

### Inclusion and exclusion criteria from the study

In order to be included in the study, it is necessary to be working as a family physician in Samsun, to agree to participate in the study voluntarily, and to complete the questionnaire. Those who did not meet at least one of the inclusion criteria were excluded from the study.

### Ethical considerations

Ethics committee approval, dated 10.02.2021 and protocol numbered GOKA/2021/3/3, was obtained from Samsun Provincial Health Directorate Scientific Research Evaluation Commission and Samsun Training and Research Hospital Non-Interventional Clinical Research Committee. Participants signed an informed consent form and were told that participation in the study was entirely voluntary and that they could leave from the study at any time. At every stage of the study, the principles of the Declaration of Helsinki were followed.

### Data collection and data collection tool

In the first part of the questionnaire used as a data collection tool, there are questions about demographic data.In the second part, the State-Trait Anxiety Inventory (STAI) scale, developed by Spielberger [20] in 1970 and adapted into Turkish by Oner and Le Compte [21] in 1983, was used for questioning anxiety. The Turkish scale can be applied to men and women over 18 years. The internal consistency and reliability of the Turkish version were found to be between 0.94 and 0.96 for the State Anxiety Inventory (STAI-S) and between 0.83 and 0.87 for the Trait Anxiety Inventory (STAI-T) in Kuder Richardson alpha reliability [21]. In this study, STAI-T Cronbach alpha was determined as 0.72, and STAI-S Cronbach alpha was 0.78.

There are ten reverse scored questions in the state anxiety scale. These are questions 1, 2, 5, 8, 10, 11, 15, 16, 19, 20. On the trait anxiety scale, the number of reverse scored questions is seven. These questions are the 21st, 26th, 27th, 30th, 33rd, 36th and 39th questions. A predetermined and unchanging value is added to the total number found. The constant value for state anxiety scale is 50 and for trait anxiety it is 35. The final value is the individual’s anxiety score. It is stated that individuals with a total anxiety score above 60 need professional help. Scale scores; Scores between 0 and 40 points are interpreted as no anxiety, scores between 41 and 60 points are interpreted as mild anxiety, and scores of 61 points and above are interpreted as severe anxiety [19, 22, 23].

### Statistical analysis

SPSS package program v23.0 was used in the analysis of the data. The suitability of the data to the normal distribution was evaluated with the Kolmogorov-Smirnov test. In the analysis of the data, descriptive statistics, in the correlation of arithmetic means, Pearson correlation test was used in the data conforming to the normal distribution, and Spearman correlation test was used in the data that did not fit the normal distribution. The t-test was used to test the significance of the difference between the two means for normally distributed data, and the One-Way ANOVA was used for multiple variances. The Mann–Whitney U test was used to test the significance of the difference between the two means for data that did not fit the normal distribution, and the Kruskal-Wallis test for multiple variances. P values below 0.05 were considered significant (*p* < 0.016 for Bonferroni correction).

## Results

Two hundred fourteen family physicians were included in the study. The majority of the participants in the study were male. Most of the family physicians were not specialist physicians. Most of them were married. The majority of family physicians did not smoke and did not have any chronic diseases. The demographic characteristics of the physicians participating in the study are given in Table 1.

**Table 1:** Demographic features

Demographic features		n	%
Gender	Female	69	32.2
	Male	145	67.8
Academic title	Family medicine specialist	22	10.3
	Family medicine research assistant	11	5.1
	Non-specialist family physician	181	84.6
Marital status	Married	186	86.9
	Single	25	11.7
	Widowed/divorced	3	1.4
Chronic disease	Present	69	32.2
	No	145	67.8
Smoking status	No	144	67.3
	Yes	65	30.4
	Left	5	2.3
Average year of practice	19.56 ± 8.52 (Min. 2–Max. 43)		
Average year of family practice	9.84 ± 4.80 (Min. 0–Max. 21)		

The number of people who had individuals at risk for COVID in their family or close relatives was higher. The rate of those whose relatives died due to COVID was approximately 16%. About 11% of the participants stated that they received psychological support before the pandemic. Although the majority of physicians stated that they had psychological difficulties during the pandemic process, the majority of them stated that they did not need psychological support. Half of the participants, as well as the majority of those who have children, stated that they had a childcare problem during the pandemic process. The majority of physicians reported that they did not experience the feeling of stigma. Family physicians reported that they were not worried that they would be a burden to their colleagues if they were quarantined. The majority of family physicians stated that they were hesitant when examining patients and that they were worried about infecting their relatives. Data on medical characteristics and social difficulties related to the pandemic process are given in Table 2.

**Table 2:** Features of medical conditions and social difficulties

		n	%
Presence of individuals at risk for COVID in relatives	Yes	130	60.7
	No	84	39.3
Being a relative who died due to COVID	Yes	34	15.9
	No	180	84.1
Experiencing mental / psychological difficulties during the pandemic process	Yes	91	42.5
	No	37	17.3
	Partial	86	40.2
Having received psychological support before the pandemic	Yes	25	11.7
	No	189	88.3
Needing psychological support during the pandemic process	I needed and got support	16	7.5
	It was needed and I did not receive support	56	26.2
	Not needed	142	66.3
Having difficulties in childcare during the pandemic process	Yes	104	48.6
	No	73	34.1
	No children	37	17.3
Feeling negative discrimination or stigma during the pandemic process	Yes	62	29.0
	No	152	71.0
Concern about increasing the workload of coworkers due to being infected or in quarantine	Yes	62	29.0
	No	152	71.0
Feeling hesitant when examining	Yes	157	73.4
	No	57	26.6
Worry about infecting relatives	Yes	207	96.7
	No	7	3.3

The trait anxiety score of the physicians was 43.40 ± 8.50, and the state anxiety score was 48.09 ± 11.55. The correlation between trait anxiety and state anxiety was positive and significant with the Spearman correlation test (*r* = 0.573, *p* < 0.001). The relationship between working time as a physician and trait anxiety was statistically insignificant with the Pearson correlation test (*r* = − 0.031, *p* = 0.648). The relationship between working time as a physician and state anxiety was also statistically insignificant (*r* = 0.012, *p* = 0.865) with the Spearman correlation test. The relationship between the duration of family practice and trait anxiety was insignificant with the Pearson correlation test (*r* = − 0.078, *p* = 0.257). The relationship between the duration of family practice and state anxiety was found to be insignificant with the Spearman correlation test (*r* = 0.030, *p* = 0.663).

Gender difference does not make a significant difference in trait and state anxiety. Trait and state anxiety were significantly higher in patients with chronic disease. Trait anxiety of family medicine specialists was significantly higher than non-specialist family physicians. Marital status, having a child, and a history of COVID-19 do not significantly effect on trait and state anxiety. The demographic features that affect the trait and state anxiety levels are given in Table 3.

**Table 3:** Demographic features affecting trait and state anxiety levels

	STAI-T	STAI-S
*Gender*		
Female	44.03 ± 7.71	48.58 ± 10.81
Male	43.10 ± 8.83	47.86 ± 11.81
*p* value	0.455*	0.669**
*Presence of chronic disease*		
Present	45.58 ± 7.76	51.48 ± 12.08
No	42.36 ± 8.66	46.48 ± 10.97
*p* value	0.009*	0.003**
*Academic title*		
Family medicine specialist^1^	48.64 ± 8.09	54.18 ± 9.25
Family medicine research assistant^2^	44.64 ± 6.54	46.82 ± 10.74
Non-specialist family physician^3^	42.69 ± 8.45	47.43 ± 11.68
*p* value	0.007^+^	0.013^++^
1–2^a^	0.398	0.191
1–3^a^	**0.005**	0.025
2–3^a^	0.792	0.984
*Marital status*		
Married	43.06 ± 8.47	47.72 ± 11.75
Single	45.56 ± 8.96	50.56 ± 10.40
Widowed/divorced	46.33 ± 2.08	50.67 ± 5.85
*p* value	0.477^+^	0.403^++^
*Having children*		
Yes	43.20 ± 8.62	48.10 ± 11.90
No	44.32 ± 7.93	48.03 ± 9.83
*p* value	0.467*	0.990**
*Being infected with COVID*		
Yes	43.30 ± 8.77	48.06 ± 11.92
No	41.50 ± 4.95	43.00 ± 2.82
*p* value	0.773*	0.484*
*Smoking status*		
Yes	44.43 ± 8.22	49.35 ± 10.66
No	43.04 ± 8.66	47.61 ± 12.10
Left	40.20 ± 6.83	45.40 ± 2.96
*p* value	0.385^+^	0.525^++^

Bold indicates* P* value < 0.05 is statistically significant

*STAI-S* State anxiety inventory,* STAI-T* Trait anxiety inventory, * independent sample t test, ** Mann–Whitney U test, ^+^ One-Way ANOVA, ^++^ Kruskall Wallis test, ^a^ Mann Whitney U test with Bonferroni correction

Those who used psychiatric treatment before the pandemic, had mental depression during the pandemic, received psychiatric treatment during the pandemic, had problems with child care during the pandemic, and those with high-risk relatives for COVID had higher trait and state anxiety. It was observed that the death of a relative due to COVID did not show any difference in anxiety scores. The anxiety scores of family physicians who thought they would be a burden to their colleagues in the possibility of being quarantined due to COVID were higher. Fear of infecting their relatives and being hesitant while examining patients were not found to be effective on anxiety scores. The characteristics of the situations that affect the trait and state anxiety levels are given in Table 4.

**Table 4:** Conditions affecting trait and state anxiety levels

	STAI-T	STAI-S
*Receiving psychiatric treatment before the pandemic*		
Yes	49.08 ± 9.28	51.60 ± 12.69
No	42.65 ± 8.12	47.62 ± 11.34
*p* value	0.001*	0.120**
*Mental depression during the pandemic process*		
Yes^1^	47.62 ± 8.31	53.76 ± 10.89
No^2^	38.03 ± 8.60	37.51 ± 9.04
Partial^3^	41.24 ± 6.337	46.64 ± 9.43
*p* value	0.001^+^	< 0.001^++^
1–2^a^	**< 0.001**	**< 0.001**
1–3^a^	**< 0.001**	**0.001**
2–3^a^	0.084	**< 0.001**
*Receiving psychiatric treatment during the pandemic process*		
I needed and got support^1^	50.50 ± 10.19	54.31 ± 13.59
It was needed and I did not receive support^2^	46.52 ± 7.59	55.84 ± 8.71
Not needed^3^	41.37 ± 7.86	44.33 ± 10.48
*p* value	< 0.001^+^	< 0.001^++^
1-2^a^	0.186	0.861
1–3^a^	**< 0.001**	**0.001**
2–3^a^	**< 0.001**	**< 0.001**
*Experiencing child care shortages in the pandemic*		
Yes^1^	45.10 ± 9.01	52.00 ± 11.26
No^2^	40.51 ± 7.28	42.55 ± 10.57
No children^3^	44.32 ± 7.93	48.03 ± 9.83
*p* value	< 0.001^+^	< 0.001^++^
1-2^a^	**0.001**	**< 0.001**
1–3^a^	0.877	0.135
2–3^a^	0.060	0.034
*Being a COVID risky relative*		
Yes	44.48 ± 7.94	49.95 ± 11.71
No	41.71 ± 9.09	45.20 ± 10.73
*p* value	0.020*	0.002**
*Death of a relative due to COVID*		
Yes	43.59 ± 7.37	47.18 ± 9.62
No	43.36 ± 8.71	48.26 ± 11.89
*p* value	0.887^*^	0.521**
*Feeling of stigma*		
Yes	44.60 ± 8.16	51.74 ± 11.91
No	42.91 ± 8.61	46.60 ± 11.10
*p* value	0.188*	0.005**
*Feeling of being a burden to co-workers in case of quarantine*		
Yes	46.84 ± 8.89	51.16 ± 12.19
No	41.99 ± 7.94	46.84 ± 11.08
*p* value	0.001*	0.014**
*Worry about infecting relatives*		
Yes	43.46 ± 8.44	48.29 ± 11.60
No	41.57 ± 10.76	42.14 ± 8.72
*p* value	0.565*	0.108**
*Hesitations when examining*		
Yes	44.17 ± 8.88	49.32 ± 12.00
No	41.26 ± 6.98	44.68 ± 9.49
*p* value	0.014*	0.012**

Bold indicates* P* value < 0.05 is statistically significant

*STAI-S* State anxiety inventory,* STAI-T* Trait anxiety inventory, * independent sample t test, ** Mann–Whitney U test, ^+^ One-Way ANOVA, ^++^ Kruskall Wallis test, ^a^ Mann Whitney U test with Bonferroni correction

## Discussion

At the beginning of the COVID pandemic, the greatest struggle was given in secondary and tertiary healthcare institutions. With the prolonged recovery period and late COVID complications, this burden has shifted to primary health care institutions. This situation increased the workload and stress of family physicians working in primary care and thus increased their perceived anxiety. In this study, perceived anxiety levels of family physicians were measured.

In our study, the trait anxiety score of primary care physicians was 43.40 ± 8.50, and the state anxiety score was 48.09 ± 11.55. In a study conducted in Indonesia in 2021, state anxiety was found to be 39.63 ± 11.54 and trait anxiety 39.42 ± 7.99 [9]. The low level of state anxiety in this study may be due to the decrease in the number of COVID cases, an increase in vaccination and immunization studies, and, as a result, being used to the pandemic situation, due to the fact that this study was conducted in the last period.

A positive significant correlation was found between trait and state anxiety. In the literature review, a positive and significant difference was found between anxiety scores in the study of Yildirim and Atas [24]. This study was conducted with dentistry students. It is about a process that requires face-to-face communication and close-range examination. As the literature supports, stressful situations increase the level of perceived anxiety.

In our study, the gender difference did not make a significant difference on these scores. In the literature, it has been seen that there are different results in this area. In the study of Hacimusalar et al. with healthcare professionals [25] and in the study of Yildirim and Atas, gender does not make a significant difference on anxiety scores [24]. In the study conducted in Italy by Naldi et al. [26], the study of Karasu et al. with healthcare workers [19], the study of Sert et al. with emergency service workers [22], and the study of Sogutlu et al. with healthcare workers [23], it was shown that gender difference creates a significant difference in anxiety scores. The fact that there are different results in this area shows that new studies should be done in this area.

In this study, marital status did not make a significant difference on state and trait anxiety, similarly, the study of Sert et al. [22] and the study of Sogutlu et al. [23] did not make a significant difference on marital status anxiety scores, and our study was compatible with the literature.

In the study, it was observed that having a child did not make a significant difference on anxiety scores. In the study conducted in Italy [26], in the study of Karasu et al. [19], and in the study of Sert et al., having a child creates a significant difference on anxiety scores [22]. The literature contradicts our work in this area. Due to the high levels of anxiety in both those who have and do not have children, we may not have found a difference in our study.

Having trouble with child care during the pandemic had a significant effect on the anxiety score. Similarly in the literature, in the study of Hacimusalar, the anxiety scores of those who had childcare difficulties were significantly higher [25]. At the same time, the anxiety score of those who have a risky relative in terms of COVID was found to be high, and in the literature, the anxiety score of those who have a risky individual at home was found to be higher [25]. These situations can be considered as effective stressor factors that increase the level of anxiety.

Presence of chronic disease had a significant effect on both trait and state anxiety, similarly in the study of Kızılkurt et al. [27] and the study of Karasu et al. [19]. Conditions such as the presence of chronic disease increase the level of perceived anxiety. Our study is compatible with the literature in these results.

Receiving psychiatric support treatment before and during the pandemic made a significant difference on anxiety scores. Similarity is observed in the study of Kızılkurt et al. [27]. It has been observed that experiencing mental depression during the pandemic process creates a significant difference on anxiety scores. Similarly, in the study of Kurt et al., depression has a significant effect on anxiety scores [28]. The effect of the fear of transmitting COVID to their relatives on anxiety was statistically insignificant, and it was seen that there was a significant difference in the study of Kurt et al. [28]. The fact that psychiatric characteristics showed similar characteristics in our study and in the literature shows that they directly affect anxiety levels.

While smoking status had a significant effect on state anxiety, it was observed that it did not have a significant effect on trait anxiety. Smoking status is not significant in the study of Sert et al. [22].Our work in this area is compatible with the literature.

## Limitations

Although Samsun is one of the largest cities in Turkey, the research was conducted in only one city. Conducting more studies in different regions, especially for primary care physicians, will confirm the data of this study and enable the establishment of newer and more advanced outcome models with new parameters to be added.

## Conclusion

Physicians working in primary health care services experience moderate anxiety during the pandemic process. Individuals who are prone to anxiety with high trait anxiety scores have higher state anxiety scores. Having a chronic disease and having a close relative at risk for COVID have a significant effect on anxiety. Anxiety scores of people who had problems with child care during the pandemic process and those who received psychological treatment before and during the pandemic were significantly higher. Our study is descriptive and our results cannot be generalized to the general population. However, the factors affecting the anxiety levels of physicians working in primary health care services should always be considered and it is important to take steps to reduce anxiety.

## Data Availability

The datasets generated and /or analyzed during the current study are not publicly available in due to ethical restrictions of the protocol having mentioned in our approved local ethical application that data will not be available for the general public but are available from the corresponding author on reasonable request.

## Abbreviations

COVID-19: Coronavirus disease 2019
SARS-CoV-2: Severe acute respiratory syndrome-coronavirus-2
WHO: World Health Organization
STAI: State-trait anxiety inventory
STAI-S: State anxiety inventory
STAI-T: Trait anxiety inventory

## References

[1] Mosheva M, Hertz-Palmor N, Dorman Ilan S, Matalon N, Pessach IM, Afek A (2020). Anxiety, pandemic-related stress and resilience among physicians during the COVID-19 pandemic. Depress Anxiety.

[2] Ashford JW, Gold JE, Huenergardt MJA, Katz RBA, Strand SE, Bolanos J (2021). MMR Vaccination: a potential strategy to reduce severity and mortality of COVID-19 illness. Am J Med.

[3] Ofei-Dodoo S, Loo-Gross C, Kellerman R, Burnout (2021). Depression, anxiety, and stress among family physicians in kansas responding to the COVID-19 pandemic. J Am Board Fam Med.

[4] Pappa S, Ntella V, Giannakas T, Giannakoulis VG, Papoutsi E, Katsaounou P (2020). Prevalence of depression, anxiety, and insomnia among healthcare workers during the COVID-19 pandemic: a systematic review and meta-analysis. Brain Behav Immun.

[5] Vilovic T, Bozic J, Vilovic M, Rusic D, Furlan SZ, Rada M (2021). Family physicians’ standpoint and mental health assessment in the light of COVID-19 pandemic—a nationwide survey study. Int J Environ Res Public Health.

[6] Berger Z, Altiery De Jesus V, Assoumou SA, Greenhalgh T (2021). Long COVID and health inequities: the role of primary care. Milbank Q.

[7] Hellemons ME, Huijts S, Bek LM, Berentschot JC, Nakshbandi G, Schurink CAM (2022). Persistent health problems beyond pulmonary recovery up to 6 months after hospitalization for COVID-19: a longitudinal study of respiratory, physical, and psychological outcomes. Ann Am Thorac Soc.

[8] Mughal F, Hossain MZ, Brady A, Samuel J, Chew-Graham CA (2021). Mental health support through primary care during and after covid-19. The BMJ.

[9] Setiawati Y, Wahyuhadi J, Joestandari F, Maramis MM, Atika A (2021). Anxiety and resilience of healthcare workers during COVID-19 pandemic in Indonesia. J Multidiscip Healthc.

[10] Leal PC, Goes TC, da Silva LCF, Teixeira-Silva F (2017). Trait vs. state anxiety in different threatening situations. Trends Psychiatry Psychother.

[11] Bandelow B, Michaelis S, Wedekind D (2017). Treatment of anxiety disorders. Dialog Clin Neurosci.

[12] Purves KL, Coleman JRI, Rayner C, Hettema JM, Deckert J, McIntosh AM, et al. The common genetic architecture of anxiety disorders. bioRxiv. 2017;203844.

[13] Sucala M, Cuijpers P, Muench F, Cardoș R, Soflau R, Dobrean A (2017). Anxiety: there is an app for that. A systematic review of anxiety apps. Depress Anxiety.

[14] Heeren A, Bernstein EE, McNally RJ (2018). Deconstructing trait anxiety: a network perspective. Anxiety Stress Coping.

[15] Carlucci L, Albaghli B, Saggino A, Balsamo M (2021). Does a fundamentalist mindset predict a state or trait anxiety? The covariate role of dogmatism. J Relig Health.

[16] Koca A, Basgul SS, Yay M (2019). Comparison of death anxiety and state-trait anxiety levels in mothers of disabled children and non-disabled children. Dusunen Adam.

[17] Turkish Statistical Institute [Internet]. [cited 2022 Dec 10]. Available from: https://data.tuik.gov.tr/Bulten/Index?p=Adrese-Dayali-Nufus-Kayit-Sistemi-Sonuclari-2020-37210#:~:text=T%C3%9 C%C4%B0K%20Kurumsal&text=T%C3%BCrkiye’de%20ikamet%20eden%20n%C3%BCfus,698%20bin%20377%20ki%C5%9Fi%20oldu.

[18] Turkish Republic Ministry of Health National Primary Health Care Services [Internet]. [cited 2022 Dec 10]. https://hsgm.saglik.gov.tr/tr/ailehekimligi/birinci-basamak-sa%C4%9Fl%C4%B1k-hizmetleri.html.

[19] Karasu F, Öztürk Çopur E, Ayar D (2022). The impact of COVID-19 on healthcare workers’ anxiety levels. J Public Health.

[20] Spielberger CD. Anxiety as an emotional state. In: Spielberger CD, editor. Anxiety: current trends in theory and research, vol. 1. 1972. pp 23–49. 10.1016/B978-0-12-657401-2.50009-5.

[21] Oner N, Le Compte A (1985). State trait inventory in handbook. Boğaziçi Univ Publ.

[22] Sert ET, Mutlu H, Kokulu K, Sarıtaş A (2020). Anxiety levels and associated factors among emergency department personnel fighting COVID-19. J Contemp Med.

[23] Söğütlü Y, Söğütlü L, Göktaş S (2021). Relationship of COVID-19 pandemic with anxiety, anger, sleep and emotion regulation in healthcare professionals. J Contemp Med.

[24] Yildirim TT, Atas O (2021). The evaluation of psychological state of dental students during the COVID-19 pandemic. Braz Oral Res.

[25] Hacimusalar Y, Kahve AC, Yasar AB, Aydin MS (2020). Anxiety and hopelessness levels in COVID-19 pandemic: a comparative study of healthcare professionals and other community sample in Turkey. J Psychiatr Res.

[26] Naldi A, Vallelonga F, Di Liberto A, Cavallo R, Agnesone M, Gonella M (2021). COVID-19 pandemic-related anxiety, distress and burnout: prevalence and associated factors in healthcare workers of North-West Italy. BJPsych Open.

[27] Kızılkurt ÖK, Güz G, Güz H, Dilbaz N (2021). State-trait anxiety levels in Turkey during COVID-19 pandemic and its relationship to somatosensory amplification. J Exp Clin Med.

[28] Kurt O, Oguzoncul AF (2020). Levels of anxiety and depression related to COVID-19 among physicians: an online cross-sectional study from Turkey. Ann Clin Anal Med.

